# An Isolated Hypogonadotropic Hypogonadism due to a L102P Inactivating Mutation of KISS1R/GPR54 in a Large Family

**DOI:** 10.1155/2019/3814525

**Published:** 2019-10-16

**Authors:** Ahmad J. Alzahrani, Azzam Ahmad, Tariq Alhazmi, Lujin Ahmad

**Affiliations:** ^1^Pediatric Endocrine Department, (A.J.A, T.A), Maternity Children Hospital, Makkah, Saudi Arabia; ^2^Umm Al-Qura University, Medical College, (A.A, L.A), Makkah, Saudi Arabia

## Abstract

KISS1R (GPR54) mutations have been reported in several patients with congenital normosmic idiopathic hypogonadotropic hypogonadism (nIHH). We aim to describe in detail nIHH patients with KISS1R (GPR54) mutations belonging to one related extended family and to review the literature. A homozygous mutation (T305C) leading to a leucine substitution with proline (L102P) was found in three affected kindred (2 males and 1 female) from a consanguineous Saudi Arabian family. This residue is localized within the first exoloop of the receptor, affects a highly conserved amino acid, perturbs the conformation of the transmembrane segment, and impairs its function. In the affected female, a combined gonadotropin administration restored regular period and ovulation and she conceived with a healthy baby boy after 4 years of marriage. We showed that a loss-of-function mutation (p.Tyr305C) in the KISS1R gene can cause (L102P) KISS1 receptor dysfunction and familial nIHH, revealing the crucial role of this amino acid in KISS1R function. The observed restoration of periods and later on pregnancy by an exogenous gonadotropin administration further support, in humans, that the KISS1R mutation has no other harmful effects on the patients apart from the gonadotropin secretion impairment.

## 1. Introduction

Hereditary isolated hypogonadotropic hypogonadism (IHH) is an uncommon disorder in pediatric and adult population; genetic causes of IHH are increasingly recognized due to the increasing number of genetic testing.

Genetic IHH is classified into two types, depending on the presence or absence of smell defect: Kallmann syndrome when associated with anosmia and normosmic IHH when normal smell is preserved.

Neuroendocrine control of the reproductive axis in humans rests with a group of neurons called GnRH (gonadotropin-releasing hormone) neurons, approximately 1500 in number and dispersed in the hypothalamus. GnRH neurons are originating from the nasal placode during embryogenesis. The hypothalamus synthesizes and releases a neurohormone, GnRH, which then travels via hypothalamic-hypophyseal portal circulation to reach the anterior pituitary, where it binds to the GnRH receptor to stimulate the synthesis and secretion of gonadotropins [1].

IHH is a heterogeneous disorder affecting one in 5000 males, with a three- to fivefold of males over females. Mutations in *KISS1/KISSR*, *TAC3/TACR3*, *GNRH1/GNRHR*, *LEP/LEPR*, *HESX1*, *FSHB*, and *LHB* are present in patients with normosmic IHH [2–4].

Several reports have showed the high potency of kisspeptin in regulating LH and FSH secretion in animals and humans [5, 6]. In 2003, two groups independently identified KISS1R as a gatekeeper of puberty: Seminara et al. [1] and de Roux et al. [7].

Inactivating mutations in KISS1R is transmitted as a recessive trait and accounts for 2–4% cases of nIHH. The *KISS1R* gene is located in locus 19p.13.3. It is also known as the *GPR54* gene, and encodes a G-protein-coupled receptor or receptor for kisspeptins. The GPR54/KISS1R protein is a transmembrane receptor made up of 398 amino acids. It translates signals from the cell surface as part of the signaling pathway for the release of GnRH. The binding of kisspeptin to these receptors in the hypothalamus stimulates the release of GnRH, which in turn stimulates gonadotropin release [8–10].

In this report, we will try to put more light in this group of patients and to show if there is any genotype-phenotype correlation by reporting an extended family of Saudi Arabian origin with KISS1R loss-of-function mutation.

## 2. Case Report

A large Saudi Arabian family with extensive intermarriage among them sought advice in different medical centers for delayed puberty and infertility. Nine of them had features of idiopathic hypogonadotropic hypogonadism (IHH).

**Figure 1:**
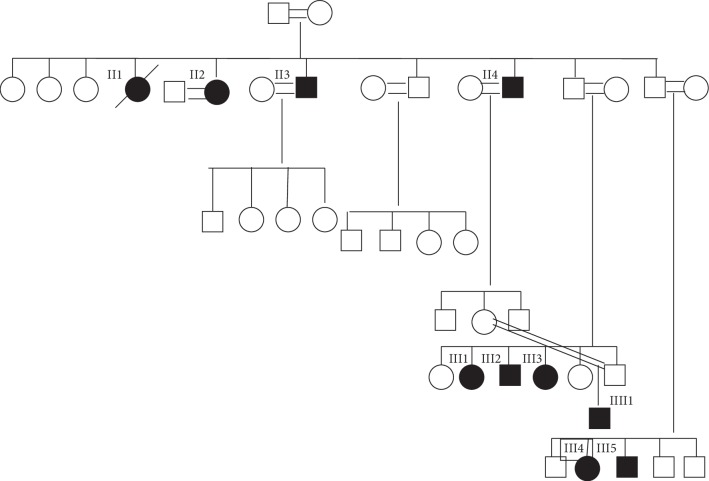
Family pedigree of the reported family: black forms indicate affected members. Circles represent female family members and squares indicate male family members. Roman numerals indicate generations and Arabic numbers indicate individuals in each generation. NL: normal.

Patients (Figure 1 (III2, III3, and IIII1)) were diagnosed and followed up at our hospital during childhood and adulthood.

Patient III3 was referred to gynecological service to continue her management after she got married. At each visit, growth measurements were recorded: height in cm and weight in kg were plotted in the CDC (Center for Diseases Control and Prevention) growth chart and the pubertal stage was assessed according to Tanner staging. Bone age (BA) was estimated according to the Greulich and Pyle method. Anterior pituitary hormone activity was evaluated by measuring the basal level of luteinizing hormone (LH), follicular-stimulating hormone (FSH), thyroid-stimulating hormone (TSH), free T4, free T3, and prolactin as well as gonadal steroid (testosterone or estrogen assay using commercial polyclonal RIA).

After informed consent was obtained, genomic DNA was extracted from white blood cells of the three patients and parents of III2 and III3 by using the standard technique.

*KISS1R* coding exons 1, 2, 3, 4, and 5 and intron-exon junctions were amplified by PCR (polymerase chain reaction) and sequenced, and *GNRHR* (*gonadotropin*-*releasing hormone receptor*) and other rare sequence variants were also analyzed as previously described [1].

Affected patients were all from consanguineous parents; there were nine affected members in this extended family (Figure 1) with different phenotypes and three of them were attending regularly to our endocrine department.

The first one (Figure 1 (III2)) was a 26-year-old male presented to the clinic with absent puberty, phallus 6 cm, testicular size 5 cc, height 173 cm, weight 65 kg, normal smell, no deafness, no skeletal defects, and no other physical abnormalities.

Laboratory workup showed low serum gonadotropins: LH 0.6 mIU/ml, FSH 3.4 mIU/ml, testosterone 0.1 ng/dl, prolactin 11 ng/ml, and normal TSH, FT3, and FT4. Repeated hormonal assay was almost similar.

Brain magnetic resonance imaging MRI showed empty sella and normal brain structure; otherwise, BA was delayed.

The patient was treated with injectable depot testosterone, gradually increasing the dose from 100 mg to 250 mg with monthly injection.

The patient had adequate pubertal development: P4, phallus 10 cm, and small testes, so the treatment was changed to human chorionic gonadotropin (hCG) (5000 IU twice weekly), and s.c. FSH injection (75 IU twice weekly) was added to enhance testicular growth and spermatogenesis; the testicular size during the last visit was 12 ml, and the patient did not appear for follow-up thereafter.

The second patient (Figure 1 (III3)) was a 17-year-old sister of the first patient who presented to our clinic with absent puberty, primary amenorrhea, normal growth parameters (height 153 cm and weight 49 kg), normal physical examination findings, normal smell, and no skeletal defects. Her hormonal assay was as follows: LH 0.9 mIU/ml, FSH 5.7 mIU/ml, estrogen 5 pg/ml, prolactin 14 ng/ml, and normal thyroid function; repeated assay was almost the same.

Brain MRI showed normal brain structure and BA was delayed.

She was treated with estrogen pills followed by oral combined estrogen/progesterone pills and after she got married she was started on human chorionic gonadotropin (hCG) together with FSH injection as the pulsatile GNRH was not available; fortunately, she got pregnant with a healthy normal boy.

The third patient (Figure 1 (IIII1)) was a 4-year-old boy, a nephew for the previous two cases, who presented with micropenis, stretched length from the pubic ramus to the top of the glans 2.2 cm, testicular volume 2.5 cc, normal physical examination findings, and normal growth parameters. Hormonal assay showed LH 0.1, FSH 0.8, and testosterone 0.2 ng/dl. Thyroid function and prolactin level were normal; the patient received a small dose of depot testosterone (50 mg IM once) and showed significant improvement in phallus size.

On reviewing the family history, it showed the following affected members (Table 1):

1. Affected aunt (II1) of the first case died at 25 years and was having no breast development, primary amenorrhea, and was married for 4 years with no pregnancy
2. A 55-year-old aunt (II2) had small breast, primary amenorrhea, married for 30 years, and no children even after pregnancy augmentation
3. A 45-year-old uncle (II3) with cryptorchidism which was treated and he is married with 5 children after infertility management
4. A 35-year-old sister (III1) with no puberty, primary amenorrhea, and married for 18 years with no pregnancy even after augmentation
5. A 30-year-old cousin (III4), lady, with no puberty, no menses, married for 5 years, and had no pregnancy even after augmentation
6. A 20-year-old cousin (III5), male, with no puberty, small testes, and small phallus

**Figure 2:**
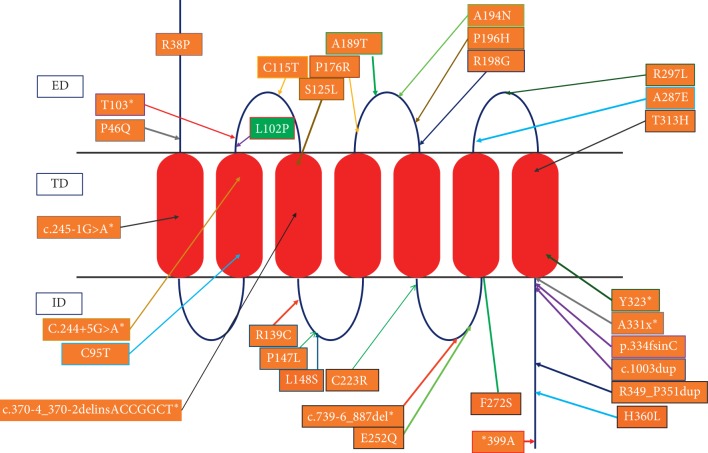
Reported mutations and its location in the KISS1 receptor identified in nCHH patients: colored in orange for previously described mutations and in green for our cases. Residues with asterisk indicate nonsense mutations, and residues without asterisk indicate missense mutations. ED: extracellular domain; TD: transmembrane domain; ID: intracellular domain.

Sequencing of the five exons of the KISS1R gene revealed homozygous mutation in exon 2 in all affected patients (III2, III3, and IIII1) as well as a heterozygous mutation in the parents of III2 and III3. A substitution of a cytosine for thymidine T305C produced a proline substitution for leucine (L102p), and this residue is localized within the first exoloop of the kisspeptin receptors (Figure 2). No mutation was identified in GNRHR or other rare related genes.

## 3. Discussion

Hereby, we described a loss-of-function mutation in the homozygous state, previously described by Yardena et al. (p.Tyr305C) in the *KISS1R* (*GPR54*) gene leading to p.Leu102Pro in the KISS1 receptor in a highly consanguineous Saudi Arabian family suffering from congenital nIHH [5]. Nine members of this family seem to be affected by this disease, but a longitudinal follow-up of three affected patients revealed inactivation of the gonadotropin axis associated with underdevelopment of external genitalia and absence of puberty.

Our reported cases differ from the previously reported similar mutation by Yardena et al. [5] in certain features: we have a larger collection (nine cases) in one family, complete absence of pubertal signs, and no cryptorchidism in the male patient (Figure 1 (III2)) with better response to treatment in the phallus size of 10 cm and the testicular growth of 12 ml compared to the previously reported cases [5] with a smaller group (three affected) in either family, partial puberty on presentation, cryptorchidism, and poor response to treatment in the male patients with phallus 7.2 cm and testes 4 ml in size which might be due to the late correction of cryptorchidism.

Functional analyses in a previous study by Yardena et al. revealed that the L102P variant led to an almost complete loss-of-function mutation, resulting in a severe phenotype corresponding to lack of pubertal development with cryptorchidism [5].

These results emphasize the role of the KISS1/KISS1R system in initiation of puberty and maintaining reproductive function through the control of GnRH secretion [10, 11].

The phenotypes of the patients appeared to be less severe in our patients as compared with the other mutations, in whom severe micropenis and cryptorchidism were reported [12], indicating that this type of mutation is less severely manifesting than other mutations.

Treating these patients demonstrated that the delayed puberty could be corrected by gonadotropin administration, which further supports the idea that the loss of KISS1R function in pituitary gonadotropic cells does not have clinically significant consequences in humans other than the gonadotropin deficiency and its consequences on health.

In our female patients, fertility was restored by gonadotropin administration where she had a healthy baby boy without miscarriages, indicating that KISS1R loss of function has no direct effect on gonadal or placental function.

The integrity of other anterior pituitary functions in the patients investigated here also shows that loss of KISS1R only affects the gonadotropic axis.

Also in Yardena et al. study, a cell surface-binding analysis revealed normal affinity of the L102P receptor for Kp10 and a small decrease in cell surface expression, which might indicate that this amino acid substitution within the first extracellular loop blocks the normal conformational change of the receptor during activation [5]. This functional analysis shows that a complete defect in the KISS1R receptor signaling results in partial gonadotropic deficiency [5]. The gonadotropic deficiency results in a more quantitative than qualitative defect in the pubertal process, and during 10 min blood sampling, low-amplitude LH pulses can still be detected in patients with KISS1R mutations. Therefore, loss-of-function mutations seem to reduce GNRH without interfering with the intrinsic GNRH pulse generator [2, 13].

One member of this family (Figure 1 (II4)) reported to have anosmia which is not associated with this type of mutation since it is not reported before to have a smell defect and most likely this is of acquired origin.

Up until mid-2019s, 32 loss-of-function mutations in the *KISS1R* (*GPR54*) gene had been described in the literature (Figure 2 and Table 2). These mutations were found to have variable clinical manifestations, varied from incomplete puberty and infertility to severe hypogonadism with microphallus and cryptorchidism in males.

In conclusion, we have identified a novel loss-of-function mutation in the KISS1R (GPR54) gene in three members of highly consanguineous families of Saudi Arabian origin associated with nIHH. This study emphasizes the important role of the KISS1/KISS1R system in maintaining gonadotropin secretion. However, little is known about hypogonadotropic hypogonadism phenotype-genotype relations in patients harboring these mutations. The current report adds to the spectrum of loss-of-function mutations in the KISS1R gene that results in loss of receptor function. Further identification of KISS1R mutations is needed to define the precise genotype-phenotype relationship.

## Figures and Tables

**Table 1:** Patients characteristics.

Patient	II1	II2	II3	III1	III2	III3	III4	III5	IIII1
Sex	F	F	M	F	M	F	F	M	M
Age, years	25	55	45	35	26	17	30	20	4
Puberty	No	No	Yes	No	No	No	No	No	Micropenis
Marriage, years	4	30	25	18	2	2	5	0	0
Children	0	0	5	0	0	1	0	0	0
Infertility treatment	Yes	Yes	Yes	Yes	No	Yes	Yes	No	No
LH					Low	Low			Low
FSH					Low	Low			Low
Treatment					Andro., FSH, hCG	hCG			Andro.

F, female; M, male; LH, luteinizing hormone; FSH, follicle-stimulating hormone; hCG, human chorionic gonadotropin; Andro., depot testosterone.

**Table 2:** Reported mutations in the KISS1R gene.

DNA mutation	Protein mutation	Functional analysis	Ethnic origin	Ref.
IVS2-4_-2del GCA ins ACCGGCT	Four different abnormal proteins	ND	Brazilian	[14]
c.305C>T	L102P	ND	Saudi Arabian	Present study
c.305C>T	L102P	IP accumulation; residual activity	Arabs	[5, 12]
c.345C>G	p.C115T	No response in luciferase	Caucasian	[12]
c.443T>C	p.L148S	IP accumulation; decreased activity	Saudi Arabian	[1]
155-bp deletion	p.247X	ND	Caucasian	[7]
c.754G>C	p.E252Q	IP accumulation	Brazilian	[14]
c.T815>C	p.F272S	IP accumulation; residual activity	Arabs	[15]
c.1001–1002insC	p.334fsinC.	ND	German	[16]
c.1079A>T	p.H360L	ND	Caucasian	[17]
c.1157G>C	p.R386P	Reduction in the rate of desensitization	Brazilian	[18]
c.667T>C	p.C223R	Ca^2+^ mobilization; low activity	Jamaican-Turkish	[19]
c.891G>T	p.R297L	Ca^2+^ mobilization; low activity	Jamaican-Turkish	[19]
c.991C>T	p.R331X	IP accumulation	Black	[1]
c.1195T>A	p.X399A	IP accumulation; low activity	Black	[1]
c.244 + 5G>A	p.?	ND	Caucasian	[12]
c.285C>G	p.Cys95Trp	No response in luciferase assays	Caucasian	[12]
c.309C>A	p.Tyr103^*∗*^	No response in luciferase assays	Caucasian	[12]
c.527C>G	p.Pro176Arg	No response in luciferase assays	Caucasian	[12]
c.860C>A	p.Ala287Glu	Shifted response in luciferase assays	Caucasian	[12]
c.113G>C	p.Arg38Pro	ND	Caucasian	[12]
c.137C>A	p.Pro46Gin	ND	Caucasian	[12]
c.374C>T	p.Ser125Leu	ND	Caucasian	[12]
c.592C>G	p.Arg198Gly	ND	Caucasian	[12]
c.969C>A/c.170T>C	p.Y323X/p.L57P	ND	Turkish	[20]
c.440C>T	p.P147L	Impaired receptor function	Japanese	[21]
c.937T>C	p.Tyr313His	ND	Portuguese	[22]
c.305T>C/c.1195T>A	p.L102P/Stop399A	No response in luciferase assays	French	[22]
c.1195T>C	p.X399R	ND	Tunisian	[23]
	R139C	Abolished membrane expression	Turkish	[24]

ND, not done; Ref, references.
